# Peripheral Expression Profiles of Glutathione Reductase and miR-144 in Patients with Atrial Fibrillation

**DOI:** 10.3390/medsci14040456

**Published:** 2026-08-05

**Authors:** Monika Różycka-Kosmalska, Mikołaj Grabarczyk, Agnieszka Śliwińska, Małgorzata Kozłowska, Marcin Kosmalski, Jerzy Krzysztof Wranicz, Izabela Szymczak-Pajor

**Affiliations:** 1 Department of Electrocardiology, Medical University of Lodz, 92-213 Lodz, Poland; monika.rozycka-kosmalska@umed.lodz.pl (M.R.-K.); jerzy.wranicz@umed.lodz.pl (J.K.W.); 2 Department of Clinical Pharmacology, Medical University of Lodz, 90-153 Lodz, Poland; mikolaj.grabarczyk@student.umed.lodz.pl (M.G.); marcin.kosmalski@umed.lodz.pl (M.K.); 3 Department of Nucleic Acid Biochemistry, Medical University of Lodz, 92-213 Lodz, Poland; agnieszka.sliwinska@umed.lodz.pl (A.Ś.); malgorzata.kozlowska@umed.lodz.pl (M.K.)

**Keywords:** atrial fibrillation, miR-144, glutathione reductase, oxidative stress, cardiology

## Abstract

**Background:** Atrial fibrillation (AF) is associated with oxidative stress and inflammation. Glutathione reductase (GR) and microRNA-144 (miR-144) may participate in redox-related pathways. This study aimed to evaluate relative *GSR* mRNA and miR-144 expression in peripheral blood samples from patients with and without AF and to explore their associations with selected clinical characteristics. **Methods:** This case–control study included 189 hospitalized adults: 95 without AF and 94 with AF. Clinical, anthropometric, biochemical, and echocardiographic data were collected for all participants. *GSR* mRNA was assessed by quantitative real-time PCR in whole blood samples, whereas circulating miR-144 expression was evaluated in serum. Group differences were assessed using nonparametric tests, correlations were evaluated using Spearman’s rank correlation coefficient, and multivariable logistic regression was used to identify variables independently associated with AF. Model stability was assessed using bootstrap internal validation. **Results:** Relative *GSR* mRNA expression was significantly lower in the AF group than in controls (0.1539 vs. 0.2058, *p* < 0.001), whereas miR-144 expression was significantly higher in patients with AF (0.3636 vs. 0.2550, *p* = 0.0183). In multivariable logistic regression adjusted for clinical confounders, higher miR-144 (OR = 1.336, *p* = 0.048) and lower *GSR* (OR = 0.343, *p* < 0.001) expression remained associated with AF. Bootstrap internal validation supported the statistical stability of the *GSR* association (optimism-corrected AUC = 0.709, bootstrapped 95% CI: 0.622–0.785), whereas miR-144 showed only a more modest and less stable association (optimism-corrected AUC = 0.598, bootstrapped 95% CI: 0.519–0.684); both signals remain exploratory and do not yet support routine clinical use as diagnostic or risk-stratification biomarkers in AF. Both markers showed exploratory correlations with selected clinical and biochemical variables. Given the cross-sectional design of the study, all reported relationships should be interpreted as statistical associations rather than as evidence of causality. The observed correlations between molecular markers and clinical parameters are hypothesis-generating and warrant further investigation.

## 1. Introduction

Atrial fibrillation (AF) is the most common sustained cardiac arrhythmia and one of the fastest-growing contributors to cardiovascular morbidity worldwide. Recent global estimates from the Global Burden of Disease 2021 project indicate that AF or atrial flutter affected approximately 52.55 million people worldwide, accounting for 8.36 million disability-adjusted life years (DALYs) and about 0.34 million deaths [1,2]. In the United States alone, AF was estimated to affect about 10.55 million adults in 2019. Contemporary reviews also emphasize the steep age dependence of the disease, with a lifetime risk of roughly 1 in 3 to 1 in 5 after the age of 45 [2,3]. Together, these data underscore that AF is not only common but also increasingly important as populations age and cardiometabolic disease becomes more prevalent [1,2,3]. Clinically, AF is defined by electrocardiographic evidence of irregular atrial activity with absent discrete P waves and an irregular ventricular response. It is commonly categorized as paroxysmal, persistent, or long-standing persistent, reflecting differences in episode duration and disease severity [2,4]. AF is far from a benign rhythm disorder—it is associated with markedly increased risks of ischemic stroke, heart failure, myocardial infarction, chronic kidney disease, cognitive decline, and death. This explains why AF has become a major target for both prevention and mechanistically informed biomarker research [2,4]. The conditions most strongly associated with the development of AF include hypertension, obesity, diabetes mellitus, obstructive sleep apnea, and various forms of thyroid dysfunction [2,4,5,6,7,8]. From a pathophysiological perspective, AF is sustained by the interaction of electrical and structural remodeling, altered calcium handling, autonomic imbalance, inflammation, and genetic or epigenetic susceptibility [4,9]. Recent reviews describe AF development and progression as dynamic processes in which shortened refractoriness, slowed and heterogeneous conduction, disturbed intracellular calcium cycling, myocyte injury, fibrosis, and inflammatory signaling progressively reinforce one another [4,9]. In this framework, oxidative stress is increasingly viewed not as a bystander but as a central integrator linking many AF risk factors to atrial arrhythmogenesis [9]. Reactive oxygen species (ROS) relevant to AF arise mainly from mitochondria, NADPH oxidases, xanthine oxidase, and uncoupled nitric oxide synthase (NOS). These sources are activated under several conditions mentioned above, which are strongly tied to AF [9]. Excess ROS can modify ion channels and redox-sensitive signaling pathways, promote abnormal calcium release, shorten atrial refractoriness, increase conduction heterogeneity, and facilitate both triggered activity and re-entry loops [9]. ROS also contribute to atrial myocyte injury, apoptosis, and interstitial fibrosis, thereby stabilizing the structural remodeling that allows AF to persist and progress [9]. Within this redox network, the glutathione system is of particular importance. Reduced glutathione (GSH) is a major intracellular antioxidant, while glutathione reductase (GR), encoded by the *GSR* gene, catalyzes the NADPH-dependent reduction of oxidized glutathione (GSSG) back to GSH and thereby helps preserve cellular redox buffering capacity. Nuclear factor erythroid 2-related factor 2 (NRF2) is a key upstream transcriptional regulator of this antioxidant program and controls multiple genes involved in glutathione synthesis and recycling, including *GCLC*, *GCLM*, and *GSR* [10,11]. Human atrial tissues show reduced atrial glutathione content in AF, and glutathione redox reactions are postulated to be among the major negatively altered pathways in AF [11,12]. In recent years, microRNAs have emerged as important post-transcriptional regulators of AF-related remodeling. Dysregulated networks of microRNAs’ interplay participate in fibrosis, inflammation, ion channel remodeling, and broader arrhythmogenic signaling in AF [13,14]. MicroRNA-144 (miR-144) is especially interesting in the context of oxidative stress and glutathione metabolism. Although the strongest mechanistic evidence comes from non-AF experimental systems, these studies are highly relevant biologically. Earlier work demonstrated that miR-144 directly represses NRF2 and that increased miR-144 expression is associated with reduced NRF2 levels, decreased glutathione regeneration, and weakened antioxidant capacity [15,16]. In an oxidative-stress model, miR-144 increased intracellular ROS and decreased the expression of glutamate–cysteine ligase catalytic subunit (GCLC), glutamate–cysteine ligase modifier subunit (GCLM), GR, and NRF2, indicating that miR-144 can downregulate the NRF2–glutathione axis at multiple levels [15]. Taken together, these previously reported findings from non-atrial experimental systems (including erythroid and neuronal cell models) and the observation that miR-144 is upregulated in AF atrial tissue make the miR-144/NRF2/GR pathway a biologically plausible, but as yet experimentally untested in AF, candidate mechanism that could plausibly contribute to redox dysregulation in AF [13,15,16]. The aim of this clinical study was to quantify the relative expression of *GSR* mRNA in whole blood and miR-144 in serum acquired from subjects with and without AF. We also aimed to investigate potential associations between their expression levels and the clinical characteristics of the examined cohorts, including anthropometric measures and basic biochemical parameters. We did not measure NRF2 expression, GR protein concentration or enzymatic activity, glutathione redox status (GSH/GSSG ratio), or direct markers of ROS burden. Consequently, the mechanistic interpretation of the miR-144/NRF2/GR axis remains hypothetical and cannot be directly inferred from our peripheral blood and serum measurements alone—a limitation explicitly acknowledged in the Section Limitations.

It should be noted at the outset that, because of the cross-sectional case–control design used here, all reported relationships between *GSR* mRNA, miR-144, and AF status are inherently associational. The present data cannot establish whether alterations in these molecular markers contribute to AF development, represent consequences of established arrhythmia, or reflect broader metabolic, inflammatory, or pharmacotherapy-related factors associated with the clinical context of AF. Causal inference would require prospective longitudinal investigations, and the present findings should therefore be regarded as hypothesis-generating rather than as evidence of mechanistic causality.

## 2. Materials and Methods

### 2.1. Study Population

This observational, case–control study enrolled 189 consecutive adult patients who were hospitalized at the Department of Electrocardiology, Medical University of Lodz, between February and April 2024 due to various internal diseases. The study group included 58 women and 131 men, with a mean age of 62.69 ± 13.85 years. The study protocol complied with the principles of the Declaration of Helsinki for research involving human participants and received approval from the Bioethics Committee of the Medical University of Lodz (approval no. RNN/01/24). Eligibility for study participation required an age between 18 and 93 years, written informed consent, and preserved verbal and logical contact enabling reliable communication. AF status was then determined based on medical history and diagnostic records. Individuals allocated to the AF-negative control group had no recorded history of AF episodes in either their medical history or available diagnostic records. Participants were excluded in the absence of written informed consent or when impaired verbal or logical contact precluded reliable communication. Additional exclusion criteria comprised the presence of secondary AF causes, including alcohol or other substance use, hyperthyroidism or hypothyroidism, signs of acute infection, significant valvular heart disease, hypertrophic or dilated cardiomyopathy, tachycardia–bradycardia syndrome, and previous cardiac surgery. Pregnant women and patients with chronic inflammatory disorders, active or prior malignancy, or systemic diseases associated with cardiac involvement such as sarcoidosis, amyloidosis, or hemochromatosis were also excluded. Moreover, individuals with suspected paroxysmal AF based on medical history, despite the lack of confirmation in diagnostic testing, were not included in the study.

### 2.2. Assessment of Baseline Clinical Characteristics in the Studied Population

All enrolled participants underwent a standardized clinical assessment that included a medical history and physical examination. Blood pressure (BP) measurements, including systolic blood pressure (SBP) and diastolic blood pressure (DBP), were obtained, and anthropometric parameters were recorded, namely body weight, height, waist circumference (WC), and hip circumference (HC). These measurements were subsequently used to calculate body mass index (BMI) and waist-to-hip ratio (WHR). Venous blood samples were then collected for laboratory analyses. The assessed parameters included fasting plasma glucose (FPG); glycated hemoglobin (HbA1c); total cholesterol (T-CH); low-density lipoprotein cholesterol (LDL-CH); high-density lipoprotein cholesterol (HDL-CH); triglycerides (TGs); total bilirubin; uric acid; urea; C-reactive protein (CRP); creatinine; and liver enzyme activity, including alanine aminotransferase (ALT), aspartate aminotransferase (AST), and gamma-glutamyl transpeptidase (GGTP). Renal function was additionally evaluated by calculating the estimated glomerular filtration rate (eGFR). The default equation available from our institutional laboratory information system at the time of blood-sample analysis (February–April 2024) was the Modification of Diet in Renal Disease (MDRD). We acknowledge that, according to the 2021 KDIGO conference report and the 2024 KDIGO Guideline for the evaluation and management of chronic kidney disease, the currently preferred creatinine-based equation in adults is the CKD-EPI 2021 ‘race-free’ equation. To address this, we additionally recomputed eGFR for all 189 participants using the published CKD-EPI 2021 coefficients. Re-analysis under CKD-EPI 2021 yielded eGFR values closely correlated with the MDRD-derived values and did not qualitatively alter any of the conclusions of the present study, including the non-significant between-group comparison of eGFR. The parallel CKD-EPI 2021-derived dataset is available from the corresponding author on reasonable request, and we recommend the use of CKD-EPI 2021 in all future studies to align with current KDIGO 2024 recommendations.

Cardiac assessment included a standard electrocardiogram (ECG) with 24 h Holter ECG monitoring and detailed rhythm analysis, in order to confirm the presence of AF (reflected in the absence of discernible and regular P waves and irregular activation of the ventricles) or exclude other cardiac arrhythmias. Transthoracic echocardiography (TTE) was also performed in all patients to determine left ventricular ejection fraction (EF). Following completion of the diagnostic work-up, the study population was divided into two groups according to AF status: patients with AF and those without AF. Comparative analyses between the groups were performed, with particular focus on baseline clinical characteristics described above, relative expression of *GSR* mRNA and miR-144, and mutual correlations between these parameters.

### 2.3. Analysis of GSR mRNA Expression

Total RNA was isolated from the whole blood samples using a Total RNA Mini Kit (A&A Biotechnology, Gdynia, Poland). RNA concentration and quality were determined spectrophotometrically employing Nanodrop 2000 (Thermo Fisher Scientific Inc., Waltham, MA, USA). Briefly, 1 μg of the purified RNA was reverse-transcribed using the High-Capacity cDNA Reverse Transcription Kit (Thermo Fisher Scientific Inc., Waltham, MA, USA), according to the manufacturer’s instructions. Quantitative real-time PCR (qRT-PCR) was then performed in duplicate using the resultant cDNA, the TaqMan Universal Master Mix (Life Technologies, Carlsbad, CA, USA), and a specific TaqMan assay (Life Technologies, Carlsbad, CA, USA) was used for the target gene GSR (ID: Hs00167317_m1). All 189 samples yielded quantifiable amplification. The intra-assay coefficient of variation (CV) for duplicate reactions was <2.5%. To ensure the absence of contamination and genomic DNA amplification, no-template controls (NTCs) and no-reverse transcription (no-RT) controls were included in each run. GAPDH (ID: Hs99999905_m1) served as the endogenous control. Gene expression levels were calculated as ΔC_t_, where ΔC_t_ = C_t (GSR)_ − C_t (GAPDH)_.

### 2.4. Analysis of miRNA-144 Expression

Total RNA was isolated from the serum samples using an RNA Isolation Kit Plasma/Serum (BioVendor, Brno, Czech Republic). The selected miR-144 expression level was quantified using RT-qPCR. Briefly, 2 µL of total RNA was subjected to reverse transcription in a 15 µL reaction volume. The reverse transcription was carried out using a TaqMan MicroRNA Reverse Transcription Kit (Applied BiosystemsTM, ThermoFisher Scientific, Waltham, MA, USA) according to the manufacturer’s manual. The RT step was conducted using a T100 Thermal Cycler (BioRad, Hercules, CA, USA). The obtained cDNA served as the template for qPCR amplification. The 10 µL qPCR mixture included 2.5 µL of diluted cDNA, 5 µL of TaqManTM Universal Master Mix II, no UNG (Applied BiosystemsTM, ThermoFisher Scientific, Waltham, MA, USA), 0.5 µL of the specific TaqManTM MicroRNA Assay (Applied BiosystemsTM, ThermoFisher Scientific, Waltham, MA, USA) for the target miRNA (ID: 477913_mir), and 2 µL of nuclease-free water. All samples were processed in two independent replicates, with intra-assay CV < 3.0%. Consistent with the mRNA protocol, no-template and no-RT controls were utilized to verify assay specificity. No samples were excluded due to qPCR failure. miR-186 (477940_mir) and miR-103 (478253_mir) were employed as endogenous reference genes for normalization.

### 2.5. Statistical Analysis

Anthropometric and biochemical characteristics, as well as miR-144 and GSR expression levels, are reported as medians with corresponding lower and upper quartiles. The non-normal distribution of these variables was confirmed using the Shapiro–Wilk test. Differences between two independent groups for continuous, non-normally distributed data were assessed using the Mann–Whitney U test. Group comparisons of categorical data, including the sex ratio, were conducted using the chi-square test. The relationships between each of the two molecular markers (relative miR-144 expression in serum and relative GSR mRNA expression in whole blood) and the continuous anthropometric and biochemical parameters listed in Section 2.2 were evaluated separately for each marker using the Spearman non-parametric correlation coefficient. Correlation analyses were performed independently within each study group (subjects without AF and patients with AF). 

No formal adjustment for multiple comparisons (e.g., Bonferroni, Holm, or Benjamini–Hochberg false discovery rate correction) was applied to the Spearman correlation analyses presented, consistent with their explicitly exploratory, hypothesis-generating design. Applying such correction across approximately 50 tests per study group would have markedly reduced statistical power and increased the risk of Type II errors, potentially obscuring weak but biologically plausible signals that may inform future, adequately powered confirmatory studies. The interpretation of nominally significant correlations therefore relies on the combined consideration of unadjusted *p*-values, effect size (R coefficient), and prior biological plausibility, whereas the resulting inflation of the family-wise Type I error rate is explicitly acknowledged in the Section Limitations.

Receiver operating characteristic (ROC) curve analysis was performed to assess the discriminatory ability of relative GSR mRNA and miR-144 expression levels to distinguish patients with AF from subjects without AF. The diagnostic performance of the analyzed markers was expressed as the area under the ROC curve (AUC). Optimal cut-off values were determined using the Youden index. Based on these cut-off values, odds ratios (ORs) with 95% confidence intervals (95% CIs) were calculated to estimate the association between altered expression of the analyzed markers and the occurrence of AF. To evaluate the stability of the diagnostic performance and correct for potential overfitting bias, internal validation of the ROC analysis was performed. A bootstrap resampling method was utilized to calculate optimism-corrected AUC estimates and generate internally validated 95% CI for the predictive models of miR-144 and GSR mRNA. Univariate and multivariable logistic regression analyses were performed to evaluate variables associated with AF status. To ensure appropriate scaling, continuous molecular variables (miR-144 and GSR mRNA) were log-transformed prior to regression modeling. The multivariable model was constructed to adjust for potential baseline confounding factors, including age, sex, renal function (eGFR), systemic inflammation (CRP), and cardiovascular pharmacotherapy (statins, beta-blockers, and renin–angiotensin–aldosterone system (RAAS) inhibitors). Medication variables were included as exploratory adjustment covariates to account for potential pharmacotherapy-related differences between groups. Because those medications have been prescribed as part of AF management or for AF-related cardiovascular comorbidities, these variables were not interpreted as causal predictors of AF. The predictive performance of each variable is reported using beta coefficients (β) with standard errors (SE), alongside ORs and their corresponding 95% confidence intervals (CIs). To construct the final multivariable model, we initially utilized a variable selection procedure—specifically a stepwise regression approach—to identify the most robust independent predictors of AF while strictly adhering to statistical thresholds for model stability. With 94 AF events in the cohort, the fully adjusted model was limited to 9 independent variables to maintain an events-per-variable (EPV) ratio of >10, which conforms to standard methodological guidelines to prevent overfitting. Although statin use was not selected by the stepwise algorithm as a statistically significant predictor, we forced its inclusion into the final model as a clinical adjustment. Because our study focuses on oxidative stress and redox-related pathways, adjusting for statins was deemed critical due to their well-documented pleiotropic, anti-inflammatory, and antioxidant properties, ensuring the observed molecular signatures were independent of this medication’s systemic effects. Conversely, HbA1c did not significantly contribute to the overall prediction of AF, aligning with the lack of baseline differences between groups, and was eliminated during the stepwise selection process. Multicollinearity among the predictor variables was assessed using the Variance Inflation Factor (VIF), with values <5 considered acceptable. Model calibration was evaluated using the Hosmer–Lemeshow goodness-of-fit test, and missing data were handled utilizing complete case analysis. All statistical calculations were performed using GraphPad Prism 8.0 software (San Diego, CA, USA). Statistical significance was established at a threshold of *p* < 0.05.

## 3. Results

### 3.1. Baseline Clinical Characteristics of the Studied Population

Table 1 presents the baseline clinical characteristics of the studied cohort. The study population comprised 189 subjects, including 95 individuals without AF and 94 patients with AF. No significant differences were observed between the groups in terms of sex distribution, age, height, body weight, BMI, WC, HC, or WHR. The groups also did not differ significantly in the prevalence of heart failure with preserved ejection fraction (HFpEF). In the AF group, permanent AF (PermAF) was the most common subtype. At the time of blood sampling, 50 patients were in AF, including 9 with paroxysmal AF (PAF). A total of 23 patients had previously undergone electrical cardioversion, including 8 patients with PAF and 2 with persistent AF (PeAF). In addition, 38 patients had a history of catheter ablation, including 23 with PAF and 2 with PeAF. With regard to renal function-related parameters, patients with AF had significantly higher urea concentrations (6.805 vs. 6.12 mmol/L, *p* = 0.0264) and creatinine levels (91.20 vs. 84.30 µmol/L, *p* = 0.0235). Although eGFR was numerically lower in the AF group than in subjects without AF, the difference was not statistically significant. No significant intergroup differences were observed for FPG, HbA1c, or uric acid levels. Lipid profile parameters, including TCH, LDL-CH, HDL-CH, and TG, were comparable between the AF and non-AF groups. Similarly, liver-related biochemical indices, including ALT, AST, GGTP, and total bilirubin, did not differ significantly between the groups, although GGTP tended to be numerically higher in patients with AF. Inflammatory status and cardiovascular parameters were also similar in both groups. CRP concentrations did not differ significantly between AF-positive and AF-negative subjects. Likewise, no significant differences were found in SBP, DBP, or EF. Medications administered in the study population are summarized in Table 2. Compared with patients without AF, those with AF more frequently received beta-blockers (BBs) (85.11% vs. 69.47%, *p* = 0.0169) and calcium channel blockers (CCBs) (23.40% vs. 10.53%, *p* = 0.0302). Antiarrhythmic treatment differed between groups, with propafenone being used markedly more often in patients with AF than in those without AF (25.53% vs. 2.11%, *p* < 0.0001). In contrast, no significant difference was observed for amiodarone use (18.09% vs. 12.63%, *p* = 0.4019). Among antithrombotic agents, acetylsalicylic acid (ASA) was used significantly less frequently in patients with AF (4.26% vs. 33.68%, *p* < 0.0001), whereas dabigatran (21.28% vs. 6.32%, *p* = 0.0055), rivaroxaban (31.91% vs. 2.11%, *p* < 0.0001), and apixaban (27.66% vs. 13.68%, *p* = 0.0282) were more commonly prescribed in the AF group. No significant between-group differences were found for angiotensin-converting enzyme inhibitors (ACEIs), angiotensin receptor blockers (ARBs), ezetimibe, statins, diuretics, mineralocorticoid receptor antagonists (MRAs), amiodarone, P2Y12 antagonists, metformin, sodium-glucose co-transporter 2 inhibitors (SGLT2is), sulfonylureas (SUs), insulin, allopurinol, vitamin K antagonists (VKAs), or angiotensin receptor/neprilysin inhibitors (ARNIs).

### 3.2. Relative GSR mRNA and miR-144 Expression in the Studied Population

**Figure 1:**
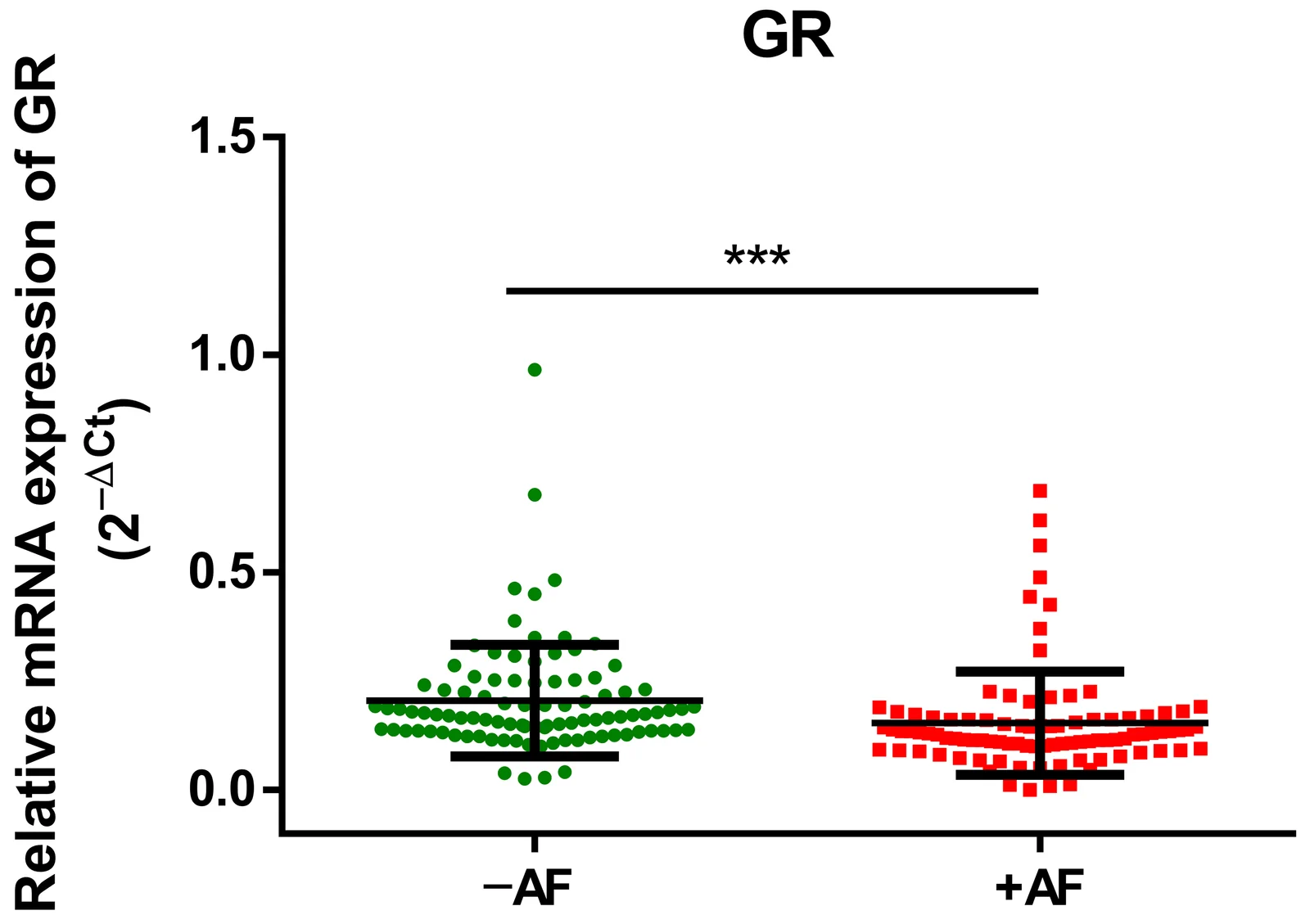
Scatter/dot plots of *GSR* relative mRNA expression levels in whole blood samples of patients with and without AF. Middle line, median; box, interquartile range; whisker, range (including outliers). *** *p* < 0.001.

**Figure 2:**
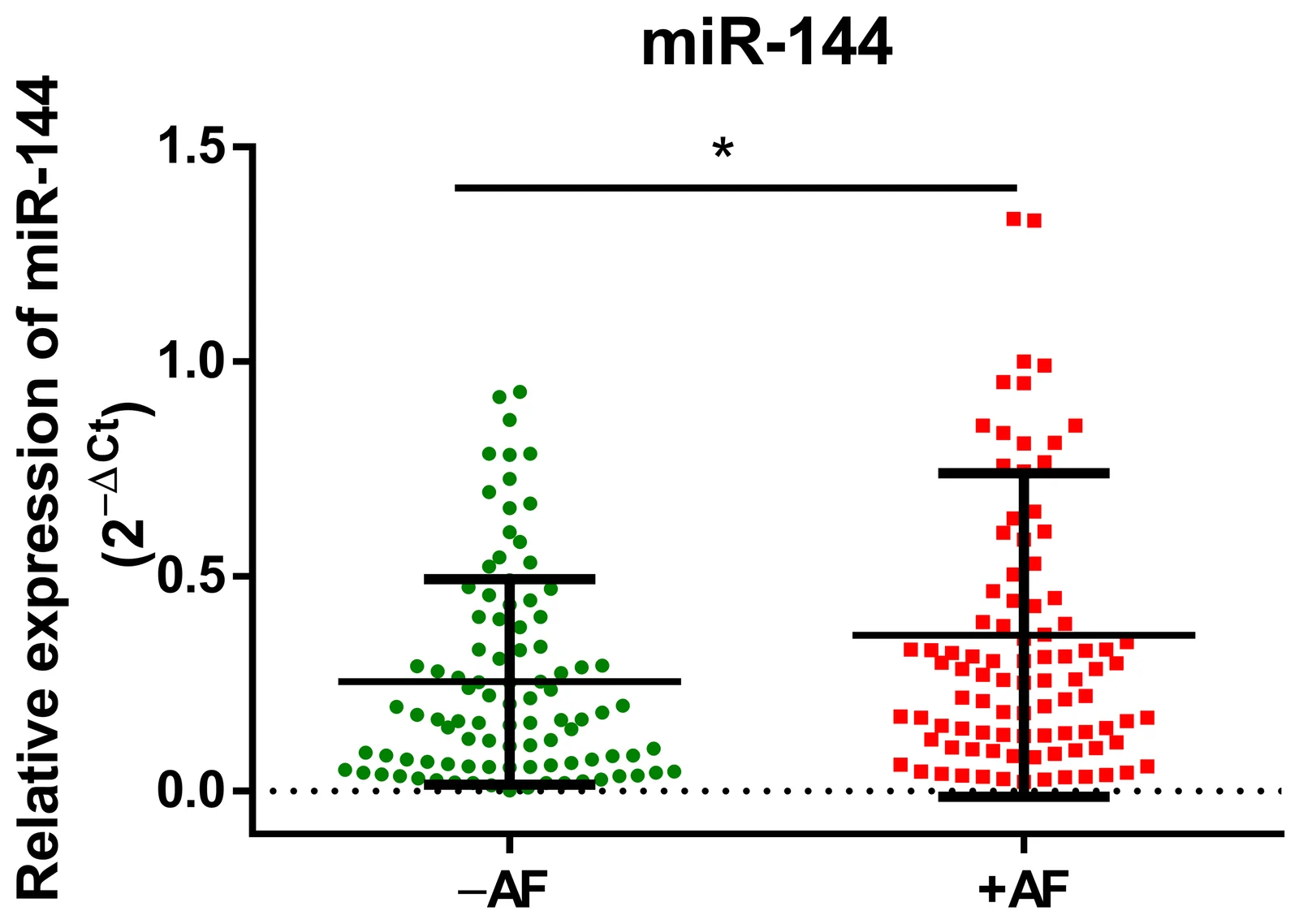
Scatter/dot plots of miR-144 relative expression levels in the serum of patients with and without AF. Middle line, median; box, interquartile range; whisker, range (including outliers). * *p* = 0.0183.

The relative mRNA expression of *GSR* was significantly lower in patients with AF when compared with the non-AF group (0.1539 vs. 0.2058, *p* < 0.001) (Figure 1). As for miR-144, the AF group displayed significantly higher relative expression than the non-AF group (0.3636 vs. 0.2550, *p* = 0.0183) (Figure 2).

### 3.3. Correlation Between Relative GSR mRNA Expression and Clinical Characteristics in the Studied Population

Table 3 presents the results of Spearman’s correlation analysis performed separately in subjects without AF and in patients with AF to evaluate the associations between relative *GSR* mRNA expression and the clinical parameters. In the group without AF, relative *GSR* mRNA expression was not significantly associated with miR-144 expression, anthropometric parameters, glucose metabolism markers, lipid profile, most liver enzymes, inflammatory markers, DBP, or EF. However, several significant correlations were identified. *GSR* mRNA expression showed a weak negative correlation with urea concentration (R = −0.2181, *p* = 0.0337) and total bilirubin level (R = −0.2364, *p* = 0.0211), as well as a weak positive correlation with eGFR (R = 0.2574, *p* = 0.0118). A borderline but non-significant negative association was observed for age (R = −0.1966, *p* = 0.0561) and SBP (R = −0.1770, *p* = 0.0862). In the AF group, relative *GSR* mRNA expression was not significantly correlated with miR-144 expression, age, anthropometric parameters, renal function indices, lipid variables, most liver-related biochemical parameters, inflammatory status, BP measurements, or EF. Significant associations were found only for HbA1c and AST. *GSR* mRNA expression showed a weak positive correlation with HbA1c (R = 0.2305, *p* = 0.0254) and a weak negative correlation with AST activity (R = −0.2621, *p* = 0.0107). No significant correlations were observed for CRP concentration or EF in patients with AF.

### 3.4. Correlation Between Relative miR-144 Expression and Clinical Characteristics in the Studied Population

Table 4 presents the results of Spearman’s correlation analysis performed separately in subjects without AF and in patients with AF to assess the relationships between relative miR-144 expression and the clinical characteristics of the studied population. In subjects without AF, relative miR-144 expression was not significantly correlated with *GSR* mRNA expression, BMI, WC, HC, WHR, urea, creatinine, glycemic indices, total cholesterol, LDL cholesterol, liver-related biochemical parameters, total bilirubin, inflammatory status, BP measurements, or EF. However, several statistically significant associations were identified. MiR-144 expression showed a moderate negative correlation with age (R = −0.3592, *p* = 0.0004), as well as weak negative correlations with body weight (R = −0.2198, *p* = 0.0324) and triglyceride concentration (R = −0.2474, *p* = 0.0156). In contrast, weak positive correlations were observed with eGFR (R = 0.3228, *p* = 0.0014) and HDL cholesterol concentration (R = 0.2255, *p* = 0.0280). Borderline but non-significant negative associations were noted for creatinine (R = −0.1892, *p* = 0.0663), FPG (R = −0.1781, *p* = 0.0842), and HbA1c (R = −0.1882, *p* = 0.0677). In the AF group, relative miR-144 expression was not significantly correlated with *GSR* mRNA expression, age, anthropometric parameters, renal function indices, glycemic parameters, HDL-CH, TG, liver enzymes, total bilirubin, CRP, BP measurements, or EF. Significant associations were observed only for lipid profile parameters. MiR-144 expression showed weak negative correlations with TCH (R = −0.2198, *p* = 0.0333) and LDL-CH (R = −0.2188, *p* = 0.0351). A borderline but non-significant positive association was observed for uric acid concentration (R = 0.1925, *p* = 0.0630).

### 3.5. Association and Discriminatory Performance of GSR mRNA and miR-144 Expression in Relation to AF in the Studied Population

**Figure 3:**
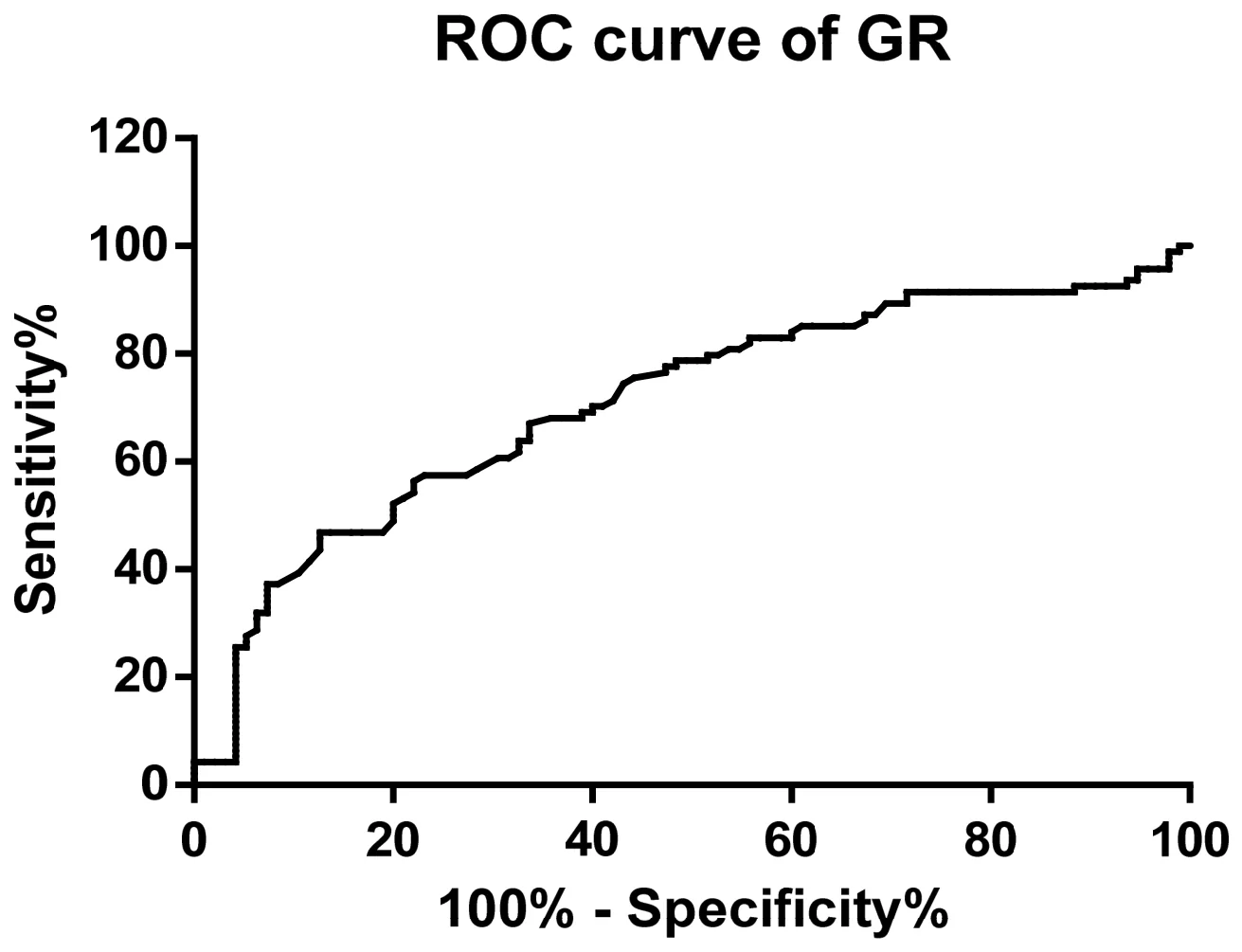
ROC curve for relative *GSR* mRNA expression in discriminating AF from non-AF subjects.

**Figure 4:**
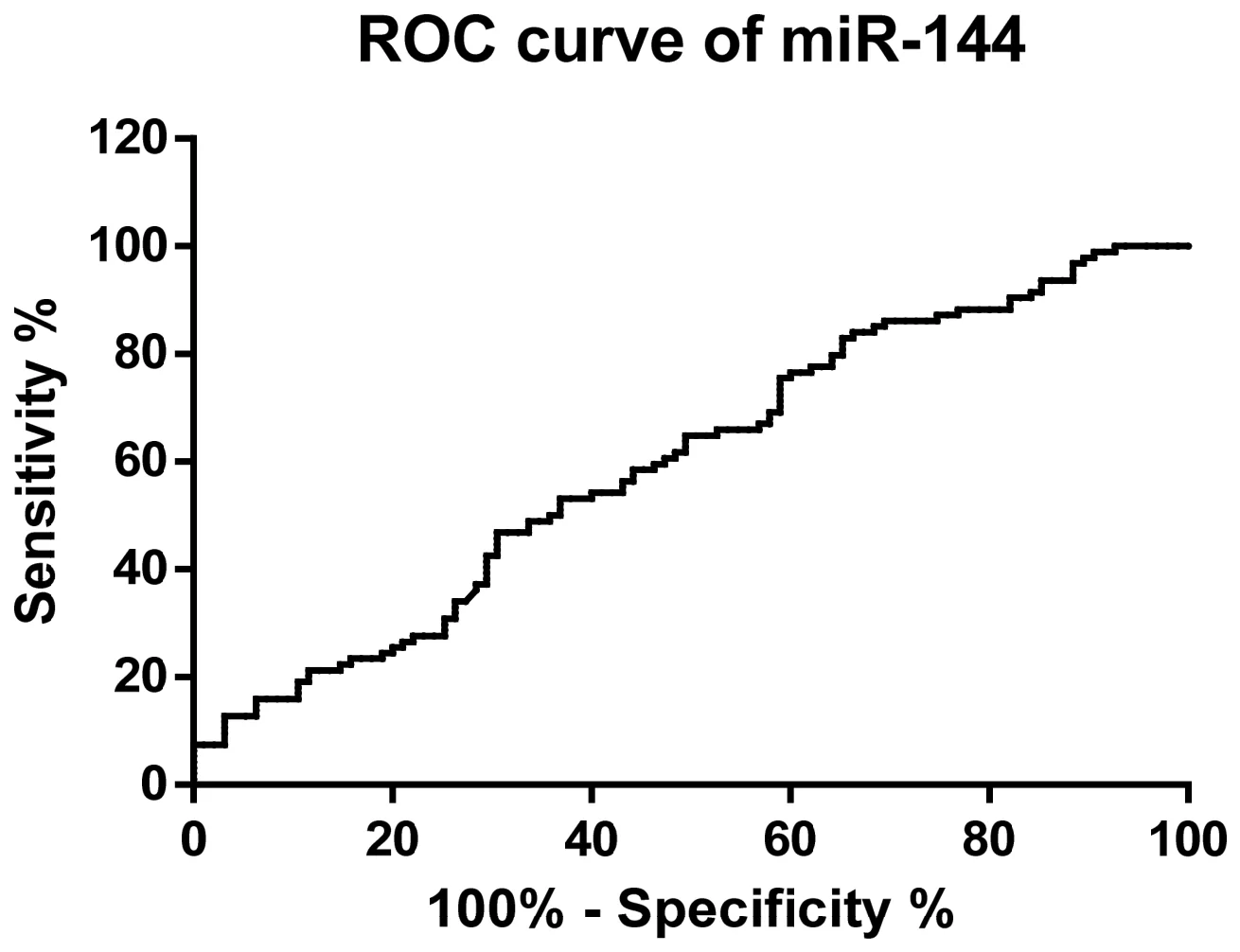
ROC curve for relative miR-144 expression in discriminating AF from non-AF subjects.

ROC curve analysis was performed to evaluate the discriminatory performance of relative *GSR* mRNA and miR-144 expression levels in distinguishing patients with AF from subjects without AF. *GSR* mRNA expression demonstrated significant discriminatory ability, with an AUC of 0.7088 (*p* < 0.001), indicating moderate accuracy (Figure 3). The optimal cut-off value determined using the Youden index was 0.1363. Based on this cut-off, *GSR* mRNA expression was significantly associated with the occurrence of AF, with an OR of 0.2232 (95% CI: 0.1191–0.4183, *p* < 0.001). For miR-144, ROC analysis also showed statistically significant discrimination between patients with and without AF. However, the discriminatory performance was weak, with an AUC of 0.5992 (*p* = 0.0185) (Figure 4). The optimal Youden-derived cut-off value was 0.0855. When this threshold was applied, miR-144 expression was significantly associated with AF occurrence, with an OR of 2.6751 (95% CI: 1.3325–5.3707, *p* = 0.0080). Internal validation using a bootstrap method was performed to assess model stability and correct for optimism bias. The optimism-corrected AUCs confirmed the stability of the ROC estimates. Specifically, the optimism-corrected AUC for *GSR* mRNA expression was 0.709 (bootstrapped 95% CI: 0.622–0.785). Although the lower bound of the GR 95% CI remained clearly above 0.50, the optimism-corrected AUC of 0.709 corresponds only to moderate discriminatory ability and remains consistent with a hypothesis-generating association rather than with clinically actionable diagnostic performance. The optimism-corrected estimate for miR-144 was 0.598, with a wider confidence interval whose lower bound (0.519) was essentially indistinguishable from the null value of 0.50, indicating a weak and statistically fragile signal that does not support standalone diagnostic use.

In the univariate logistic regression analysis (Table 5), higher expression of miR-144 was significantly associated with increased odds of AF (OR = 1.432, 95% CI: 1.097–1.869, *p* = 0.008). Advanced age (OR = 1.023, *p* = 0.035) and the use of beta-blockers (OR = 2.511, *p* = 0.012) were also identified as significant risk factors. Conversely, elevated *GSR* mRNA expression demonstrated a strong inverse association with the presence of AF (OR = 0.364, 95% CI: 0.207–0.641, *p* < 0.001).

To determine whether the molecular markers independently predicted AF status, a multivariable model was constructed to adjust for baseline demographics, renal function, systemic inflammation, and medication use (Table 6). In the fully adjusted model, both miR-144 and *GSR* mRNA maintained statistical significance. Higher miR-144 expression (Adjusted OR: 1.336, 95% CI: 1.002–1.781, *p* = 0.048) and higher *GR* expression (Adjusted OR = 0.343, 95% CI: 0.188–0.624, *p* < 0.001) remained associated with AF. Notably, clinical covariates that were significant in the univariate assessment—such as age and beta-blocker use—were attenuated and lost statistical significance in the multivariable model, indicating that the associations of both molecular markers with AF status were statistically independent of the included demographic, renal, inflammatory, and pharmacological covariates. However, the modest magnitude and wide confidence intervals of these adjusted ORs—particularly for miR-144, whose adjusted lower-bound 95% CI approximates the null value of 1.0—together with the moderate and weak optimism-corrected AUC estimates (~0.71 and ~0.60, respectively), indicate that both associations should be interpreted as exploratory statistical signals rather than as clinically actionable discriminatory performance.

## 4. Discussion

Oxidative stress has been proposed to play a role in both the initiation and progression of AF, although the temporal and causal directionality of this relationship—whether oxidative stress contributes to AF development, is a consequence of the arrhythmia, or reflects a bidirectional interaction—remains an open question [4,9]. Earlier experimental and translational work has associated excessive ROS generation with arrhythmogenic substrate formation and has suggested that oxidative stress disturbs calcium handling, shortens atrial refractoriness, impairs sodium-channel and gap-junction function, and promotes inflammation and fibrosis. Some authors have further proposed that the dominant sources of ROS may evolve during the natural history of AF, with NADPH oxidases suggested to be particularly relevant in earlier phases and mitochondrial dysfunction becoming increasingly important as AF persists and structural remodeling advances [9,17]. The present study cannot directly inform these mechanistic models, as discussed in detail in the Section Limitations.

Within this broader redox framework, the glutathione system appears especially relevant. GSH is one of the principal intracellular antioxidant buffers, and GR, encoded by the *GSR* gene, is essential for restoring GSH from its oxidized form, thereby maintaining cellular redox homeostasis [18,19]. Carnes et al. demonstrated that left atrial glutathione content was significantly lower in patients with either paroxysmal or persistent AF than in controls without AF. In the same study, incubation of atrial myocytes acquired from AF patients with the glutathione precursor N-acetylcysteine increased L-type calcium current. Simultaneously, experimental glutathione depletion in canine atria reduced atrial contractility. The authors also showed an inverse relationship between atrial glutathione content and S-nitrosylation of calcium-channel proteins, suggesting that glutathione depletion may contribute to the nitroso-redox modifications involved in electrical remodeling [11]. In the present study, patients with AF exhibited significantly lower relative *GSR* mRNA expression in peripheral blood than subjects without AF. This observation is consistent with prior reports of impaired glutathione-related antioxidant defense in AF [11], although the cross-sectional design of the present analysis does not allow us to determine whether reduced *GSR* mRNA expression contributes to AF pathophysiology, results from chronic arrhythmia burden, or reflects systemic metabolic, renal, inflammatory, or pharmacotherapy-related factors that co-occur with AF. The fact that GR is responsible for the regeneration of reduced glutathione from its oxidized form provides a biologically plausible—but mechanistically unproven in the present setting—explanation for why lower *GSR* mRNA levels might be associated with diminished glutathione-dependent redox buffering in AF [11,19]. Importantly, ROC analysis further supported the relevance of *GSR* mRNA expression in relation to AF status. *GSR* mRNA relative expression showed significant and moderate discriminatory ability in distinguishing AF-positive from AF-negative subjects, with an AUC of 0.7088. Moreover, when the ROC-derived cut-off value was applied, higher *GSR* mRNA expression was associated with significantly lower odds of AF occurrence. Conversely, reduced *GSR* mRNA expression below the ROC-derived cut-off was associated with higher unadjusted odds of AF in this cohort. However, this association should be interpreted strictly as an exploratory statistical link within the present case–control sample, and it does not currently support the use of *GSR* mRNA relative expression as a clinical rule-in/rule-out marker for AF. The bootstrap internal validation provides additional support for the robustness of the GR signal. The optimism-corrected AUC for *GSR* mRNA remained virtually unchanged compared with the apparent ROC estimate, and its confidence interval remained clearly above the null value of 0.50. This suggests that the moderate discriminatory performance of *GSR* mRNA was not driven solely by apparent-sample optimism. By contrast, the optimism-corrected AUC for miR-144 remained close to the original estimate but showed a wider confidence interval approaching the null threshold. Therefore, miR-144 appears to be associated with AF status, but its standalone discriminatory utility is limited and less stable than that of *GSR* mRNA. This interpretation is also in line with other human translational studies suggesting that glutathione-related imbalance is linked not only to AF presence but also to AF burden and progression. In a cohort of 1439 patients undergoing coronary angiography, Tahhan et al. reported that a more oxidized plasma glutathione redox potential was associated with both prevalent and incident AF, with stronger associations for chronic than paroxysmal AF [20]. Similarly, proteomic analyses of human left atrial appendage tissue identified glutathione redox reactions among the pathways altered in AF and during AF progression [12]. Taken together with our findings, these data are broadly compatible with the concept that disruption of glutathione-related antioxidant defense may accompany both the occurrence and increasing severity of AF. However, given that all currently available clinical evidence—including the present study—comes from cross-sectional or observational designs, a causal role of glutathione-pathway disruption in AF initiation or progression cannot be inferred from these data, and prospective longitudinal studies are needed to clarify the temporal relationship. However, individual components of the glutathione system may not behave uniformly, depending on the disease’s phenotype. In human atrial myocardium samples acquired from patients undergoing cardiac surgery, Anderson et al. showed that monoamine oxidase (MAO) was a major determinant of redox balance and that MAO, myocardial total glutathione, and glutathione peroxidase (GPx) were associated with increased risk of postoperative atrial fibrillation (POAF). Notably, GR was not associated with POAF risk in that cohort [21]. A similar assessment conducted by Watt et al. yielded consistent findings, showing that the transcriptome of left atrial tissue differed significantly between patients who developed POAF and those who did not. They found that patients with POAF exhibited higher levels of proinflammatory transcripts, particularly interleukin-6 (IL-6), and lower expression of antioxidant effectors, including GR and superoxide dismutase 2 (SOD2). Expression of glutathione synthetase (GSS) also showed a downward trend, although this effect did not reach statistical significance [22]. This suggests that glutathione-related antioxidant dysfunction in AF may not necessarily be captured by every enzymatic component to the same extent in every clinical setting. By contrast, Rubanenko et al. found in patients undergoing coronary artery bypass grafting that postoperative oxidative-stress markers, including lower glutathione, GPx, and GR, were strongly associated with new-onset POAF. According to multivariable analysis, GR concentration ≤ 2.99 mmol/g hemoglobin was associated with higher odds of POAF in that cohort [23]. Taken together, these three studies suggest that glutathione-pathway abnormalities are relevant to AF biology, but the strength and exact location of the signal may vary according to phenotype, timing of sampling, and whether atrial tissue or circulating markers are examined. The overall clinical context should also be considered when interpreting the present findings. In the examined cohort, AF and non-AF groups did not differ significantly in age, sex distribution, anthropometric indices, lipid profile, inflammatory status, blood pressure, or EF. The main baseline biochemical differences were limited to higher urea and creatinine concentrations in patients with AF, whereas eGFR did not differ significantly between groups. Thus, the observed reduction in peripheral *GSR* mRNA relative expression cannot be simply attributed to broad differences in obesity-related or inflammatory clinical characteristics between the groups. Nevertheless, the higher urea and creatinine levels in the AF group suggest that renal-function-related factors may still be associated with systemic redox imbalance and should be considered as potential modifiers—but not causal determinants—of peripheral molecular profiles. The finding of higher miR-144 expression in subjects with AF is also of particular interest and warrants further investigation. As with *GSR* mRNA, this association does not establish whether elevated miR-144 contributes to AF pathophysiology, represents a downstream consequence of atrial remodeling, or is driven by systemic factors associated with AF and its comorbidities. Although direct AF-specific mechanistic evidence for miR-144 remains limited, several original studies support the biological plausibility of a link between miR-144 and glutathione-dependent antioxidant defense [13,15,16]. Sangokoya et al. showed that miR-144 directly represses NRF2, reduces antioxidant response element-driven transcription, and impairs glutathione regeneration during oxidative stress [16]. In parallel, van den Berg et al. identified miR-144-3p among the upregulated microRNAs in human atrial tissue from patients with paroxysmal and persistent AF, linking it to structural-remodeling signatures [13]. In the present study, miR-144 expression was significantly higher in patients with AF. However, its discriminatory performance was weaker than that of *GSR* mRNA. ROC analysis showed an AUC of 0.5992, indicating only limited ability to distinguish patients with AF from subjects without AF. Nevertheless, after applying the ROC-derived cut-off, higher miR-144 expression was associated with increased odds of AF occurrence. Therefore, miR-144 is statistically associated with AF status in this cohort, but its weak discriminatory performance (AUC ≈ 0.60) does not currently support any meaningful standalone clinical application as a diagnostic or risk-stratification biomarker in AF. Circulating-miRNA studies suggest that the behavior of miR-144 may be more complex and context-dependent. In the exploratory plasma study by Kiyosawa et al., miR-144-5p showed a negative correlation with the CHA_2_DS_2_ -VASc score and a positive relationship with clinical performance of catheter ablation during follow-up, suggesting that lower plasma miR-144-5p may characterize a higher-risk and potentially more profibrotic AF phenotype [24]. Likewise, Kiliszek et al. identified miR-144-3p among candidate serum miRNAs associated with AF recurrence after ablation in the discovery phase, but this signal was not confirmed in the full validation cohort [25]. Taken together, these data indicate that the literature on miR-144 in AF remains heterogeneous and may depend on the biological compartment studied, the clinical phenotype of AF, and the specific miR-144 strand analyzed (miR-144-3p vs. miR-144-5p). In this context, our observation of higher peripheral-blood miR-144 expression in AF remains biologically coherent with the prior mechanistic literature. However, this coherence should be regarded as hypothesis-restoring rather than mechanistic-explanatory: the referenced miR-144–NRF2 interaction was demonstrated in non-atrial cell systems (SH-SY5Y neurons [15] and erythroid progenitors [16]) and has not been directly verified in atrial tissue, in patients with AF, or in the present cohort. In the absence of direct measurements of NRF2 expression, GR protein or activity, GSH/GSSG ratio, or ROS load, our data therefore provide statistical support—rather than mechanistic proof—for the conjecture that elevated miR-144 might be associated with diminished GR-dependent redox buffering in AF. Causal interpretation of any miR-144–NRF2–GR regulatory relationship in AF would require dedicated mechanistic studies.

At the same time, our results suggest that this relationship is more complex than a simple one-to-one regulatory axis. Despite the opposite group-level patterns of *GSR* mRNA and miR-144 relative expression, we did not observe a significant direct correlation between the two markers within either study group. This lack of direct association confirms that GR levels are heavily influenced by other systemic factors, rather than being solely dictated by a straightforward linear regulatory relationship with miR-144 in whole blood. As demonstrated in our analyses, *GSR* mRNA expression is modulated by broader metabolic and renal-function-related factors such as eGFR, urea, HbA1c, and transaminase activity. The lack of direct correlation between *GSR* mRNA and miR-144 expression may also be explained by the distinct biological compartments evaluated in this study. *GSR* mRNA was quantified in whole blood to capture the intracellular antioxidant transcriptional response of circulating cells. In contrast, miR-144 was quantified in serum to evaluate its profile as a circulating, extracellular signaling molecule. Because these markers were assessed in different compartments—intracellular versus systemic cell-free—their expression levels reflect related, but physically and biologically distinct, regulatory pools. In subjects without AF, *GSR* mRNA expression correlated negatively with urea and total bilirubin and positively with eGFR, whereas in patients with AF, it correlated positively with HbA1c and negatively with AST activity. By contrast, miR-144 expression in subjects without AF correlated negatively with age, body weight, and triglyceride concentration and positively with eGFR and HDL-CH. In the AF group, significant miR-144 correlations were limited to weak negative associations with TCH and LDL-CH. This pattern suggests that peripheral-blood expression of *GSR* mRNA and miR-144 may be influenced by overlapping but non-identical biological processes, including metabolic stress, inflammation, and cardiac functional status. However, given that several of the nominally significant correlations reported in Table 3 and Table 4—particularly those involving small correlation coefficients (|R| < 0.27) and borderline *p*-values in the range 0.02–0.03—would not survive formal Bonferroni or Benjamini–Hochberg false-discovery-rate correction for the approximately 50 independent tests performed per study group, these specific correlational findings should be regarded as strictly hypothesis-generating and require confirmation in adequately powered, pre-registered prospective cohorts before any clinical or biological interpretation can be considered. If an miR-144–NRF2–GR regulatory axis operates in AF, it may be context-dependent, cell-type-specific, and partly obscured in whole blood, where the measured signal reflects mixed circulating cell populations rather than atrial myocardium alone. We frame this statement explicitly as a mechanistic conjecture anchored to prior non-AF experimental findings rather than as a conclusion demonstrated in the present clinical cohort. Verifying whether such an axis functions in atrial myocardium will require direct measurements of NRF2 and downstream targets in atrial tissue, which were not performed in the present study and constitute an explicit limitation (see the Section Limitations).

The differences in pharmacotherapy between groups also deserve consideration when interpreting the molecular findings. Observed disparities were expected and likely reflect standard AF management, particularly the markedly higher use of direct oral anticoagulants, BBs, CCBs, and propafenone, together with lower use of ASA in the AF group [2,26]. However, pharmacological treatment may partly shape the broader inflammatory and oxidative systemic conditions. Therefore, the observed differences in *GSR* mRNA and miR-144 expression should be interpreted in the context of both AF itself and the treatment/comorbidity pattern associated with it [7,9]. The univariate and multivariable logistic regression analyses provide an additional perspective on the relationship between the analyzed molecular markers and AF status. It must be emphasized that these regression models describe statistical associations adjusted for selected covariates and do not establish a causal directionality between molecular markers and AF. The persistence of *GSR* mRNA and miR-144 associations with AF status after multivariable adjustment should therefore be interpreted as evidence of statistical independence from the included confounders, not as proof of a mechanistic or causal role [27]. In univariate analysis, higher miR-144 expression was associated with increased odds of AF, whereas higher *GSR* mRNA expression showed a strong inverse association with AF (Table 5). This direction of effect is biologically consistent with the proposed redox-related mechanism [9]. Among clinical variables, age and BB use were also associated with AF in univariate analysis. The association with age is expected, as age is one of the strongest epidemiological determinants of AF and reflects cumulative exposure to structural, metabolic, and inflammatory remodeling. The univariate association between beta-blocker use and AF should be interpreted differently. BBs are commonly prescribed in patients with AF for ventricular rate control and are also frequently used in cardiovascular comorbidities that predispose to AF. Therefore, BB use in this case–control setting may represent treatment indication, previous clinical decision-making, or the underlying cardiovascular disease burden rather than a diagnostic predictor or causal determinant of AF [26,27]. In the multivariable model adjusted for age, sex, eGFR, CRP, statin use, BB use, and RAAS inhibitor use, the associations of both molecular markers with AF status persisted. Higher miR-144 expression remained positively associated with AF, whereas higher *GSR* mRNA expression remained inversely associated with AF. While the miR-144–AF association persisted in the multivariable model, its relevance was marginal (adjusted OR: 1.336, 95% CI: 1.002–1.781). With the lower bound of the confidence interval resting practically at 1.0, this multivariable signal is consistent with the modest ROC-derived discriminatory performance of miR-144 (AUC ≈ 0.60). In parallel, the *GSR* mRNA–AF association also remained significant after multivariable adjustment (Adjusted OR: 0.343, 95% CI: 0.188–0.624), but its adjusted effect size and moderate bootstrap-validated AUC of ≈0.71 likewise indicate an exploratory statistical association rather than clinically actionable discriminatory performance. Taken together, both markers should be interpreted as AF-associated peripheral molecular signals whose independent effects, although statistically significant, are of modest magnitude and require external validation before any clinical translation can be considered.

### Limitations

This study has several limitations that should be acknowledged. First and foremost, the cross-sectional case–control design and the single-time-point assessment of molecular markers do not allow any conclusions about causality or temporal directionality. Therefore, although lower *GSR* mRNA expression and higher miR-144 expression were observed in patients with AF in this cohort, it cannot be determined whether these molecular alterations contribute to AF development, result from the arrhythmia itself, or reflect broader clinical, metabolic, inflammatory, or pharmacotherapy-related factors that differ between groups. This limitation applies equally to all reported associations, including those observed in univariate and multivariable regression analyses and in ROC-based discriminatory estimates. To establish whether *GSR* mRNA downregulation and miR-144 upregulation actively precede, accompany, or follow AF onset would require prospective longitudinal studies with incident AF cases, ideally combined with serial biomarker sampling and atrial tissue correlation. Until such studies are available, both markers should be regarded exclusively as AF-associated molecular signals rather than as mechanistic drivers of the disease [27]. Second, the sample size was relatively modest, which may have limited statistical power, particularly for subgroup analyses and for detecting weaker associations between molecular markers and clinical variables. Although the AF and non-AF groups did not differ significantly in most baseline anthropometric, metabolic, lipid, inflammatory, and cardiovascular parameters, patients with AF had higher urea and creatinine concentrations. These renal-function-related differences may have influenced oxidative-stress-related molecular profiles independently of AF status and may therefore represent a potential source of residual confounding. The participants also differed in selected medication classes, particularly those related to standard AF management, including BBs, CCBs, propafenone, ASA, and direct oral anticoagulants. Although these differences were clinically expected, pharmacological treatment may still affect systemic inflammatory, metabolic, or oxidative pathways and should be considered when interpreting the observed differences in *GSR* mRNA and miR-144 expression. Moreover, while circulating miRNAs offer valuable systemic insights, serum miR-144 assays are particularly sensitive to red blood cell rupture. Although grossly hemolyzed samples were avoided, the potential influence of microscopic, undetected hemolysis on serum miR-144 determinations cannot be entirely ruled out and should be considered when interpreting these extracellular expression levels. In addition, the analysis was based on peripheral blood and serum samples rather than atrial tissue. As a result, the measured *GSR* mRNA and miR-144 expression levels may not fully reflect molecular processes occurring directly in the atrial myocardium, where AF substrate formation takes place. Furthermore, the present study evaluated only relative expression of *GSR* mRNA and miR-144. We did not perform any of the following measurements, which would be required to substantiate a mechanistic miR-144/NRF2/GR axis in AF: (i) NRF2 mRNA or NRF2 protein expression; (ii) GR protein concentration or GR enzymatic activity; (iii) total/oxidized/reduced glutathione levels (GSH, GSSG, and GSH/GSSG ratio); (iv) downstream oxidative-stress markers such as malondialdehyde, 8-iso-prostaglandin F2α, 4-hydroxynonenal adducts, protein carbonylation, or direct ROS quantification; (v) NRF2 nuclear translocation or NRF2–antioxidant response element binding assays; or (vi) functional rescue experiments (e.g., NRF2 activation, GR supplementation, and N-acetylcysteine administration). Consequently, any mechanistic interpretation of a miR-144/NRF2/GR axis in AF derived from the present data remains explicitly indirect and hypothetical. The miR-144–NRF2 interaction referenced throughout this manuscript was demonstrated in non-cardiac experimental systems [15,16], and direct extrapolation of that interaction to AF—or to the clinical associations reported here—should be made only with the caveat that the present study is, by design, not equipped to test it. Future confirmatory studies should incorporate paired atrial-tissue NRF2/GR/GSH measurements, ideally combined with functional perturbation of the axis, before any mechanistic claim can be substantiated in the AF context.

Another limitation concerns the ROC and OR analyses. The optimal cut-off values for *GSR* mRNA and miR-144 expression were derived from the same study cohort in which their discriminatory performance and ORs were assessed. Although bootstrap internal validation was used to correct for optimism in ROC-based estimates, this approach does not replace external validation in an independent cohort. Therefore, these thresholds should be considered exploratory and require further validation before they can be interpreted as clinically useful cut-off points. Finally, although individuals with suspected but unconfirmed PermAF were excluded at the enrollment stage based on available medical history and diagnostic records, the possibility that some control subjects harbored truly asymptomatic, brief, or otherwise undocumented paroxysmal AF episodes cannot be completely excluded. Because the applied diagnostic workup relied on a standard 12-lead ECG, a single 24 h Holter recording, and review of prior medical documentation, it is inherently insensitive to very short-lasting, low-burden, or predominantly nocturnal paroxysmal AF episodes, which—as documented in contemporary AF screening literature—may remain entirely undetected despite the absence of clinical suspicion [3,26]. Consequently, a small proportion of subjects assigned to the control group may, by chance, have been misclassified with respect to true AF status, representing an unavoidable limitation of the present case–control design. This residual misclassification would be expected to bias the observed between-group differences in GSR mRNA and miR-144 expression toward the null hypothesis, i.e., to attenuate rather than inflate the reported associations [27]. For these reasons, the present results should be interpreted as hypothesis-generating and require confirmation in larger, prospectively designed, multicenter studies incorporating more detailed phenotyping, external validation of ROC-derived thresholds, atrial tissue analyses, and mechanistic assessment of oxidative-stress-related pathways. A further limitation is the potential for residual confounding. While our multivariable model adjusted for available demographic and clinical variables, we did not fully account for other specific factors known to influence oxidative stress and circulating miRNA profiles, such as smoking status, the detailed extent of coronary artery disease, and comprehensive clinical classifications of heart failure severity. Although baseline proxies for obesity (BMI, waist circumference) and diabetes burden (HbA1c, fasting plasma glucose) were statistically comparable between the groups, the influence of unmeasured cardiovascular comorbidities cannot be entirely excluded. Consequently, the independent predictive value of the reported biomarkers should be interpreted with appropriate caution. A major methodological limitation of this study concerns the pre-analytical handling and quantification of circulating miR-144. Because miR-144 is highly abundant in erythrocytes as part of the erythroid miR-144/451 cluster, its serum levels are remarkably sensitive to even minimal, visually undetectable hemolysis. Furthermore, our analytical protocol did not incorporate exogenous spike-in controls (such as cel-miR-39) to actively monitor RNA extraction efficiency, nor did we perform formal spectrophotometric or molecular (e.g., miR-23a/miR-451a ratio) assessments of hemolysis. Given the modest effect size observed for miR-144 in our cohort, we cannot completely rule out the possibility that uncontrolled pre-analytical factors, rather than the underlying arrhythmia alone, may have influenced the observed differences between the AF and non-AF groups. Consequently, the findings regarding serum miR-144 must be interpreted with caution, and future investigations must incorporate strict hemolysis monitoring and spike-in controls to validate these exploratory results.

Methodological limitations also extend to the eGFR-estimating equation and to multiplicity adjustment of the correlation analyses, specifically, the following:

- eGFR equation: All eGFR values reported in Table 1, in the correlation tables (Table 3 and Table 4), and in the multivariable regression models (Table 5 and Table 6) were derived using the MDRD equation, which was the routine in-house equation at our institution at the time of laboratory analysis. The 2024 KDIGO guideline currently recommends the CKD-EPI 2021 creatinine-based ‘race-free’ equation as the preferred eGFR estimator in adults. We additionally recomputed eGFR under CKD-EPI 2021 for all 189 participants and confirmed that reanalysis did not qualitatively alter any of the present conclusions (parallel dataset available from the corresponding author on reasonable request). Future confirmatory studies should preferentially use CKD-EPI 2021 eGFR values to align with current guideline recommendations.
- Multiplicity adjustment of correlation analyses: The Spearman correlation analyses reported in Table 3 and Table 4 comprise approximately 50 hypothesis tests per study group (each marker correlated with ~25 anthropometric, biochemical, and clinical parameters within each AF stratum). Because no formal correction for multiple comparisons (Bonferroni, Holm, or Benjamini–Hochberg false discovery rate) was applied, a non-negligible proportion of the nominally significant correlations—particularly those with small effect sizes (|R| < 0.27) and borderline *p*-values in the 0.02–0.03 range—is expected to reflect chance-level false-positive findings rather than genuine biological associations. These correlational signals therefore warrant a strictly hypothesis-generating interpretation and must be confirmed in pre-specified, adequately powered prospective cohorts before any clinical or mechanistic conclusion can be drawn.

## 5. Conclusions

Patients with AF exhibited significantly lower relative *GSR* mRNA expression in peripheral blood and significantly higher relative miR-144 expression in serum than subjects without AF. Consistent with the cross-sectional design of the study, these findings should be interpreted strictly as statistical associations between molecular markers and AF status and support the concept that AF is associated with an altered peripheral molecular profile compatible with—but not proof of—impaired antioxidant defense and dysregulation of the glutathione-related redox system. ROC curve analysis further indicated that *GSR* mRNA expression had moderate ability to discriminate between AF-positive and AF-negative subjects, whereas miR-144 showed only weak discriminatory performance. ORs analysis based on ROC-derived cut-off values confirmed that higher *GSR* mRNA expression was associated with lower odds of AF occurrence, while higher miR-144 expression was associated with increased odds of AF. Although no direct correlation between *GSR* mRNA and miR-144 expression was observed within either study group, both markers showed exploratory associations with selected clinical and biochemical variables, suggesting that their expression may be influenced by broader metabolic, renal-function-related, and lipid-related factors rather than by AF alone. In patients with AF, *GSR* mRNA expression was related to HbA1c and AST activity, whereas miR-144 expression was associated with TCH and LDL-CH levels. Taken together, although both markers reached statistical significance, these findings should be regarded as exploratory and hypothesis-generating rather than as evidence of clinically actionable discriminatory performance of either *GSR* mRNA or miR-144 in AF. Between the two analyzed markers, *GSR* mRNA appears to have greater potential for distinguishing patients with AF from subjects without AF, while miR-144 should be interpreted primarily as an AF-associated molecular signal rather than as a strong standalone discriminatory marker. Importantly, the absence of a significant intra-group correlation between *GSR* mRNA and miR-144 expression in our data does not establish—nor refute—the existence of an miR-144–NRF2–GR regulatory axis in AF, because neither NRF2 expression nor downstream components of this axis (GR protein, GR activity, GSH/GSSG ratio, and ROS load) were directly measured in the present study. Any interpretation of our findings as direct evidence of, or as mechanistic refutation of, such an axis is therefore not supported by the present data and should be regarded as explicitly hypothetical. In summary, our results indicate that peripheral-blood *GSR* mRNA downregulation and serum miR-144 upregulation are statistically associated with AF status. In the absence of direct measurement of NRF2, GR protein or activity, glutathione redox status, or ROS burden, these statistical associations should be interpreted as compatible with—but not as proof of—the hypothesis that oxidative-stress-related alterations accompany AF. The mechanistic pathway that has been hypothesized for this association in the prior literature (an miR-144–NRF2–GR axis) remains untested in the present cohort and would require dedicated functional studies in AF-relevant tissues before any mechanistic claim can be made. However, given the potential for residual confounding by unmeasured cardiovascular comorbidities and lifestyle factors, the independence of these biomarkers must be interpreted with caution. Before any clinical translation of these exploratory signals as AF-associated biomarkers can be considered, the present findings must be confirmed and externally validated in larger, independent, prospectively designed multicenter cohorts. Future studies should ideally combine atrial tissue analyses, direct assessment of glutathione redox status, and prospective longitudinal follow-up to clarify whether the observed associations reflect a causal role of GR or miR-144 in AF pathophysiology, secondary manifestations of established arrhythmia, or broader metabolic and renal-function-related factors, and to determine whether GR or miR-144 can meaningfully serve as biomarker targets in AF.

## Figures and Tables

**Table 1:** Basic clinical characteristics in patients with and without AF.

Parameter	Patients Without AF (n = 95)	Patients with AF (n = 94)	*p*
Sex [% of Female]	35.79	25.53	0.1263
HFpEF [%]	71.57	64.89	0.4310
PeAF [%]	0	14.89	**<0.0001**
PermAF [%]	0	52.12	**<0.0001**
PAF [%]	0	32.97	**<0.0001**
Duration of all AF [years]	0	1.58 [0; 2]	**<0.0001**
Age [years]	63 [46; 72]	67 [60; 72]	0.0595
Height [cm]	170 [168; 178]	174 [166; 180]	0.4497
Body weight [kg]	82 [72; 95]	85.5 [77; 100]	0.0682
BMI [kg/m^2^]	28.13 [24.61; 31.56]	29.15 [26.34; 31.86]	0.0807
WC [cm]	100 [90; 108]	104 [93.75; 110.3]	0.1035
HC [cm]	104 [98; 112]	105.5 [102; 112]	0.2838
WHR	0.94 [0.86; 1.00]	0.96 [0.8875; 1.020]	0.1947
Urea [mmol/L]	6.12 [4.85; 7.62]	6.805 [5.25; 8.743]	**0.0264**
Creatinine [µmol/L]	84.30 [68.90; 101.2]	91.20 [78.15; 108.5]	**0.0235**
eGFR [ml/min/1.73 m^2^]	75.70 [55.50; 93.60]	69.40 [56.78; 89.95]	0.0958
FPG [mmol/L]	5.68 [5.19; 6.54]	5.745 [5.23; 6.52]	0.9667
HbA1c [%]	5.7 [5.4; 6.1]	5.7 [5.4; 6.1]	0.6965
Uric acid [µmol/L]	318.4 [258.5; 403]	337.3 [299.6; 395.6]	0.1088
TCH [mmol/L]	4.6 [3.57; 5.35]	4.305 [3.533; 5.305]	0.8469
LDL-CH [mmol/L]	2.7 [1.88; 3.2]	2.44 [1.98; 3.12]	0.2723
HDL-CH [mmol/L]	0.99 [0.78; 1.45]	1.045 [0.86; 1.385]	0.5072
TG [mmol/L]	1.67 [1.01; 2.37]	1.52 [0.97; 2.183]	0.3621
ALT [U/L]	22.65 [16.6; 32.3]	23.35 [18.08; 30.18]	0.8562
AST [U/L]	25.1 [21.2; 30.8]	25.85 [21.45; 31.55]	0.7376
GGTP [U/L]	29.2 [20.20; 45.00]	33.35 [24.25; 46.73]	0.0943
Total bilirubin [µmol/L]	12.8 [9.4; 17.50]	13.80 [10.38; 17.90]	0.1368
CRP [mg/L]	2 [0.8; 4.7]	2.3 [0.975; 4.650]	0.5463
SBP [mmHg]	127 [112; 140]	130 [115; 140]	0.6361
DBP [mmHg]	78 [70; 82]	80 [70; 83.25]	0.9502
EF [%]	56.00 [45; 60]	55 [43; 60]	0.4901

Abbreviations: AF, atrial fibrillation; ALT, alanine aminotransferase; AST, aspartate aminotransferase; BMI, body mass index; CRP, c-reactive protein; DBP, diastolic blood pressure; EF, ejection fraction; eGFR, estimated glomerular filtration rate; FPG, fasting plasma glucose; GGTP, gamma-glutamyl transpeptidase; HbA1c, glycated hemoglobin; HC, hip circumference; HDL-CH, high-density lipoprotein cholesterol; HFpEF, heart failure with preserved ejection fraction; LDL-CH, low-density lipoprotein cholesterol; PAF, paroxysmal atrial fibrillation; PeAF, persistent atrial fibrillation; PermAF, permanent atrial fibrillation; SBP, systolic blood pressure; TCH, total cholesterol; TG, triglycerides; WC, waist circumference; WHR, waist-to-hip ratio. Data are expressed as medians [quartile 1; quartile 3]. *p*-values were assessed using the Mann–Whitney U test except for sex, HFpEF, and AF subtype distribution, for which the chi-square test was used (data are expressed as percentages). The bolded results indicate statistically significant differences.

**Table 2:** Drugs given to patients with and without atrial fibrillation (AF).

Medication	Patients Without AF (n = 95)	Patients with AF (n = 94)	*p*
ACEI	49 (51.58%)	51 (54.26%)	0.7125 *
ARB	20 (21.05%)	20 (21.28%)	0.9699 *
Ezetimibe	5 (5.26%)	5 (5.32%)	0.7583 **
Statins	52 (54.74%)	57 (60.64%)	0.4116 *
BB	66 (69.47%)	80 (85.11%)	**0.0169** **
CCB	10 (10.53%)	22 (23.40%)	**0.0302** **
Diuretic	37 (38.95%)	49 (52.13%)	0.0689 *
MRA	35 (36.84%)	46 (48.94%)	0.0930 *
Propafenone	2 (2.11%)	24 (25.53%)	**<0.0001** ***
Amiodarone	12 (12.63%)	17 (18.09%)	0.4019 **
ASA	32 (33.68%)	4 (4.26%)	**<0.0001** ***
P2Y12 antagonist	13 (13.68%)	5 (5.32%)	0.0871 **
Metformin	22 (23.16%)	21 (22.34%)	0.8934 *
SGLT2i	26 (27.37%)	24 (25.53%)	0.7747 *
SU	1 (1.05%)	6 (6.38%)	0.0573 ***
Insulin	6 (6.32%)	4 (4.26%)	0.3802 ***
Allopurinol	12 (12.63%)	20 (21.28%)	0.1644 **
Dabigatran	6 (6.32%)	20 (21.28%)	**0.0055** **
Rivaroxaban	2 (2.11%)	30 (31.91%)	**<0.0001** ***
Apixaban	13 (13.68%)	26 (27.66%)	**0.0282** **
VKA	2 (2.11%)	7 (7.45%)	0.0821 ***
ARNI	10 (10.53%)	4 (4.26%)	0.0879 ***

Abbreviations: ACEI, angiotensin-converting enzyme inhibitor; ASA, acetylsalicylic acid; ARNI, angiotensin receptor–neprilysin inhibitor; ARB, angiotensin II receptor blocker; BB, beta-blocker; CCB, calcium channel blocker; MRA, mineralocorticoid receptor antagonist; SGLT2i, sodium-glucose cotransporter-2 inhibitor; SU, sulfonylurea; VKA, vitamin K antagonist. The data are expressed as the number of observations with a given variable variant (n) and its corresponding percentage (%). The bolded results indicate statistically significant differences. * *p*-value was assessed using the chi-square test. ** *p*-value was assessed using the Yates continuity-corrected chi-square test. *** *p*-value was assessed using Fisher’s exact test.

**Table 3:** Univariate correlations of whole blood level of *GSR* mRNA relative expression and clinical parameters in patients with and without AF.

Parameter	Patients Without AF		Patients with AF	
	R	*p*	R	*p*
miR-144	−0.0986	0.3418	−0.0613	0.5571
Age [years]	−0.1966	0.0561	0.0302	0.7726
Body weight [kg]	0.0794	0.4438	0.0723	0.4886
BMI [kg/m^2^]	−0.0083	0.9361	0.0786	0.4517
WC [cm]	−0.0006	0.9954	0.0040	0.9693
HC [cm]	0.0094	0.9278	0.0663	0.5253
WHR	0.0335	0.7470	0.0159	0.8795
Urea [mmol/L]	**−0.2181**	**0.0337**	0.0377	0.7186
Creatinine [µmol/L]	−0.1667	0.1064	−0.0776	0.4572
eGFR [mL/min/1.73 m^2^]	**0.2574**	**0.0118**	0.1271	0.2223
FPG [mmol/L]	−0.0447	0.6671	−0.0119	0.9090
HbA1c [%]	−0.0826	0.4264	**0.2305**	**0.0254**
Uric acid [µmol/L]	−0.0987	0.3411	−0.1078	0.3011
TCH [mmol/L]	−0.0333	0.7489	−0.0391	0.7084
LDL-CH [mmol/L]	0.0363	0.7268	0.0073	0.9446
HDL-CH [mmol/L]	−0.0755	0.4672	0.0869	0.4052
TG [mmol/L]	−0.0067	0.9487	−0.0845	0.4182
ALT [U/L]	0.0082	0.9372	0.0027	0.9797
AST [U/L]	−0.0200	0.8473	**−0.2621**	**0.0107**
GGTP [U/L]	−0.0959	0.3554	0.0041	0.9684
Total bilirubin	**−0.2364**	**0.0211**	−0.1370	0.1881
CRP [mg/L]	−0.0017	0.9872	0.0557	0.5937
SBP [mmHg]	−0.1770	0.0862	0.0926	0.3747
DBP [mmHg]	0.0128	0.9022	0.1051	0.3132
EF [%]	0.1101	0.2880	0.0813	0.4361

Abbreviations: AF, atrial fibrillation; ALT, alanine aminotransferase; AST, aspartate aminotransferase; BMI, body mass index; CRP, C-reactive protein; DBP, diastolic blood pressure; EF, ejection fraction; eGFR, estimated glomerular filtration rate; FPG, fasting plasma glucose; GGTP, gamma-glutamyl transpeptidase; HbA1c, glycated hemoglobin; HC, hip circumference; HDL-CH, high-density lipoprotein cholesterol; LDL-CH, low-density lipoprotein cholesterol; miR-144, microRNA 144; *R*, Spearman’s correlation coefficient; SBP, systolic blood pressure; TCH, total cholesterol; TG, triglycerides; WC, waist circumference; WHR, waist-to-hip ratio. The bolded results indicate significant correlations as assessed by the Spearman correlation method.

**Table 4:** Univariate correlations of serum level of miR-144 relative expression and clinical parameters in patients with and without AF.

Parameter	Patients Without AF		Patients with AF	
	R	*p*	R	*p*
*GSR* mRNA	−0.0986	0.3418	−0.0613	0.5571
Age [years]	**−0.3592**	**0.0004**	0.1198	0.2502
Body weight [kg]	**−0.2198**	**0.0324**	−0.0019	0.9856
BMI [kg/m^2]^	−0.0982	0.3435	0.0635	0.5433
WC [cm]	−0.1282	0.2156	0.0929	0.3731
HC [cm]	−0.1058	0.3075	0.0552	0.5973
WHR	−0.0975	0.3470	0.1019	0.3285
Urea [mmol/L]	−0.1668	0.1061	0.0054	0.9588
Creatinine [µmol/L]	−0.1892	0.0663	−0.0255	0.8072
eGFR [mL/min/1.73 m^2^]	**0.3228**	**0.0014**	−0.0608	0.5603
FPG [mmol/L]	−0.1781	0.0842	−0.0626	0.5492
HbA1c [%]	−0.1882	0.0677	0.0396	0.7049
Uric acid [µmol/L]	−0.0413	0.6912	0.1925	0.0630
TCH [mmol/L]	0.0664	0.5225	**−0.2198**	**0.0333**
LDL-CH [mmol/L]	0.1225	0.2370	**−0.2188**	**0.0351**
HDL-CH [mmol/L]	**0.2255**	**0.0280**	−0.0887	0.3953
TG [mmol/L]	**−0.2474**	**0.0156**	−0.0227	0.8279
ALT [U/L]	0.0068	0.9484	0.0331	0.7515
AST [U/L]	0.0897	0.3871	0.0739	0.4793
GGTP [U/L]	−0.1145	0.2694	−0.0736	0.4808
Total bilirubin [µmol/L]	−0.0008	0.9941	0.0613	0.5570
CRP [mg/L]	−0.0476	0.6469	0.0114	0.9135
SBP [mmHg]	0.0056	0.9569	−0.1564	0.1321
DBP [mmHg]	0.0706	0.4968	−0.0481	0.6454
EF [%]	0.1137	0.2728	0.0228	0.8272

Abbreviations: AF, atrial fibrillation; ALT, alanine aminotransferase; AST, aspartate aminotransferase; BMI, body mass index; CRP, C-reactive protein; DBP, diastolic blood pressure; EF, ejection fraction; eGFR, estimated glomerular filtration rate; FPG, fasting plasma glucose; GGTP, gamma-glutamyl transpeptidase; *GSR* mRNA, messenger RNA transcribed from the glutathione reductase gene; HbA1c, glycated hemoglobin; HC, hips circumference; HDL-CH, high-density lipoprotein cholesterol; LDL-CH, low-density lipoprotein cholesterol; R, Spearman’s correlation coefficient; SBP, systolic blood pressure; TCH, total cholesterol; TGs, triglycerides; WC, waist circumference; WHR, waist-to-hip ratio. The bolded results indicate significant correlations as assessed by the Spearman correlation method.

**Table 5:** Univariate logistic regression analysis of molecular and clinical variables associated with AF status.

Predictor Variable	Univariate β (SE)	Univariate OR [95% CI]	Univariate *p*
miR-144	**0.359 (0.136)**	**1.432 [1.097–1.869]**	**0.008**
*GSR* mRNA	**−1.010 (0.289)**	**0.364 [0.207–0.641]**	**<0.001**
Sex [% of F]	−0.486 (0.319)	0.615 [0.329–1.149]	0.128
Age [years]	**0.023 (0.011)**	**1.023 [1.002–1.045]**	**0.035**
eGFR [mL/min/1.73 m^2^]	−0.011 (0.006)	0.990 [0.977–1.002]	0.097
CRP [mg/L]	0.003 (0.034)	1.003 [0.938–1.073]	0.921
Statins	0.242 (0.295)	1.274 [0.714–2.271]	0.412
BB	**0.921 (0.365)**	**2.511 [1.227–5.139]**	**0.012**
RAAS inhibitors	0.318 (0.292)	1.375 [0.776–2.436]	0.276

Abbreviations: AF, atrial fibrillation; BB, beta-blocker; CI, confidence interval; CRP, C-reactive protein; eGFR, estimated glomerular filtration rate; *GSR* mRNA, messenger RNA transcribed from the glutathione reductase gene; miR-144, microRNA-144; OR, odds ratio; RAAS, renin–angiotensin–aldosterone system; SE, standard error; β, regression coefficient. The bolded results indicate significant associations as assessed by univariate logistic regression analysis.

**Table 6:** Multivariable logistic regression analysis evaluating variables associated with AF status.

Predictor Variable	Multivariable β (SE)	Multivariable OR [95% CI]	Multivariable *p*
miR-144	**0.290 (0.147)**	**1.336 [1.002–1.781]**	**0.048**
*GSR* mRNA	**−1.071 (0.305)**	**0.343 [0.188–0.624]**	**<0.001**
Sex [% of F]	−0.485 (0.373)	0.616 [0.296–1.278]	0.193
Age [years]	0.007 (0.015)	1.007 [0.977–1.038]	0.672
eGFR [mL/min/1.73 m^2^]	−0.007 (0.009)	0.993 [0.975–1.011]	0.416
CRP [mg/L]	−0.026 (0.039)	0.974 [0.902–1.052]	0.501
Statins	−0.213 (0.362)	0.808 [0.398–1.643]	0.557
BB	0.735 (0.433)	2.086 [0.892–4.875]	0.090
RAAS inhibitors	−0.027 (0.345)	0.974 [0.495–1.914]	0.938

Abbreviations: AF, atrial fibrillation; BB, beta-blocker; CI, confidence interval; CRP, C-reactive protein; eGFR, estimated glomerular filtration rate; *GSR* mRNA, messenger RNA transcribed from the glutathione reductase gene; miR-144, microRNA-144; OR, odds ratio; RAAS, renin–angiotensin–aldosterone system; SE, standard error; β, regression coefficient. The bolded results indicate significant associations as assessed by multivariable logistic regression analysis.

## Data Availability

The datasets used and/or analyzed during the current study are available upon reasonable request from the corresponding author. The data are not publicly available due to privacy.

## Abbreviations

The following abbreviations are used in this manuscript:

ACEI	Angiotensin-converting enzyme inhibitors
AF	Atrial fibrillation
ALT	Alanine aminotransferase
ARBs	Angiotensin receptor blockers
ARNIs	Angiotensin receptor/neprilysin inhibitors
ASA	Acetylsalicylic acid
AST	Aspartate aminotransferase
AUC	Area under the ROC curve
BBs	Beta-blockers
BP	Blood pressure
BMI	Body mass index
CCBs	Calcium channel blockers
CIs	Confidence intervals
CRP	C-reactive protein
DBP	Diastolic blood pressure
eGFR	Estimated glomerular filtration rate
ECG	Electrocardiogram
EF	Left ventricular ejection fraction
FPG	Fasting plasma glucose
GCLC	Glutamate–cysteine ligase catalytic subunit
GCLM	Glutamate–cysteine ligase modifier subunit
GGTP	Gamma-glutamyl transpeptidase
GPx	Glutathione peroxidase
GR	Glutathione reductase
GSH	Reduced glutathione
HbA1c	Glycated hemoglobin
HDL-CH	High-density lipoprotein cholesterol
HC	Hip circumference
HFpEF	Heart failure with preserved ejection fraction
LDL-CH	Low-density lipoprotein cholesterol
MDRD	Modification of Diet in Renal Disease
miR-144	MicroRNA-144
MRA	Mineralocorticoid receptor antagonist
NOS	Nitric oxide synthase
NRF2	Nuclear factor erythroid 2-related factor 2
ORs	Odds ratios
PAF	Paroxysmal atrial fibrillation
PeAF	Persistent atrial fibrillation
PermAF	Permanent atrial fibrillation
POAF	Postoperative atrial fibrillation
SE	Standard errors
SGLT2is	Sodium-glucose co-transporter 2 inhibitors
SUs	Sulfonylureas
RAAS	Renin–angiotensin–aldosterone system
ROC	Receiver operating characteristic
ROS	Reactive oxygen species
SBP	Systolic blood pressure
TCH	Total cholesterol
TG	Triglycerides
TTE	Transthoracic echocardiography
VKA	Vitamin K antagonist
WHR	Waist-to-hip ratio
